# Supplementary material for: Tonian carbonaceous compressions indicate that Horodyskia is one of the oldest multicellular and coenocytic macro-organisms
Source: Commun Biol. 2023 Apr 12;6:399. doi: 10.1038/s42003-023-04740-2 (PMC10097871; doi:10.1038/s42003-023-04740-2)
Supplement: Supplementary file 2 — Supplementary Information [file 42003_2023_4740_MOESM2_ESM.pdf]

Supplementary Information for

Tonian carbonaceous compressions indicate that *Horodyskia* is one of the oldest multicellular and coenocytic macro-organisms

Guangjin Li, Lei Chen*, Ke Pang*, Qing Tang, Chengxi Wu, Xunlai Yuan, Chuanming Zhou, Shuhai Xiao

* Corresponding authors. E-mail address: kepang@nigpas.ac.cn (K.P.); leichen@nigpas.ac.cn (L.C.)

This file includes:

Supplementary Note 1: Geological setting

Supplementary Figure 1

Supplementary Figure 2

Supplementary References

Supplementary Note 1: Geological setting

Late Mesoproterozoic to early Neoproterozoic successions are widely distributed in the eastern margin of North China Craton, including the Huainan and Huaibei regions, and western Shandong, southern Liaoning, and southern Jilin provinces[1]. Recent studies suggest that a large rift basin developed on the eastern North China Craton during the late Mesoproterozoic to the early Neoproterozoic era[2,3], and it was subsequently staggered by the Tan-Lu fault[4], resulting in the sporadic and discontinuous exposure of late Mesoproterozoic to early Neoproterozoic outcrops along the eastern margin of North China Craton (Fig. 1a). Carbonaceous compression fossils in this study were collected from the Shiwangzhuang Formation of the Tumen Group in western Shandong Province (Fig. 1b) and the Jiuliqiao Formation of the Feishui Group in the Huainan region (Fig. 1c).

The Tumen Group in western Shandong Province (Fig. 1b) has been the focus of paleontological investigation in recent years[5-7]. It is divided into the Heishanguan, Erqingshan, Tongjiazhuang, Fulaishan, and Shiwangzhuang formations, in ascending order[8]. The Heishanguan Formation is composed of purple shales and glauconitic quartz sandstones containing gravels. The Erqingshan Formation mainly consists of glauconitic quartz sandstones, thin-bedded limestones, and calcareous shales. The Tongjiazhuang Formation is mainly composed of quartz sandstones, pebbly sandstones, and yellow-green shales. In places (e.g., at the studied section at Baishicun[9]), the Tongjiazhuang Formation sits directly on the Archean Taishan Group or Paleoproterozoic gneissose granite, whereas the Heishanguan and Erqingshan formations are absent. The Fulaishan Formation mainly consists of fine-grained sandstones, siltstones with some interbedded shales, and marlstones. The Shiwangzhuang Formation mainly consists of limestones, stromatolite limestones, argillaceous limestones, and dolomites. The Shiwangzhuang Formation contains a diverse assemblage of macrofossils preserved as carbonaceous compressions, including discoidal, tomaculate, and cylindrical fossils such as *Chuaria*, *Tawuia*, *Sinosabellidites*, *Protoarenicola*, and *Pararenicola*, as well as putative macroalgae with large cells, such

as *Anqiutrichoides constrictus* and *Eosolena magna*[6]. The Heishanguan, Erqingshan, and Fulaishan formations were deposited in littoral facies, the Tongjiazhuang Formation was deposited in shallow marine facies, and the Shiwangzhuang Formation was deposited in restricted platform facies[9].

The depositional age of the Tumen Group is constrained to be late Mesoproterozoic to early Neoproterozoic. Abundant organic-walled microfossils, including the potential late Mesoproterozoic to early Neoproterozoic index taxon *Trachyhystrichosphaera*[5], and two youngest detrital zircon ages from the Tongjiazhuang Formation[10,11], suggest that the depositional age of the Tongjiazhuang Formation is constrained between ~1060 Ma and ~720 Ma. The youngest detrital zircon age from the Fulaishan Formation[12] and macroscopic carbonaceous compression fossils from the Shiwangzhuang Formation, including *Chuaria*, *Tawuia, Sinosabellidites, Protoarenicola,* and *Pararenicola*, constrain the depositional age of the Fulaishan and Shiwangzhuang formations to be ~850–720 Ma[6]. The depositional age of the underlying Heishanguan and Erqingshan formations are not well constrained. They may be deposited in the late Mesoproterozoic based on correlation with equivalent strata from adjacent areas, i.e., the Huainan and Huaibei regions and southern Liaoning Province[1].

The Feishui Group and the underlying Huainan Group located in the Huainan region constitute a sedimentary mega-sequence that is bounded by unconformities above and below (Fig. 1c). These two groups contain abundant carbonaceous compression macrofossils and organic-walled microfossils, and they have been targets of paleontological investigation since the late 1970s[13-15]. The Huainan Group represents a fining-upward sequence and is composed of the Caodian, Bagongshan, and Liulaobei formations, in ascending order. The Caodian Formation consists of pebbly sandstones and overlies the metamorphosed Mesoproterozoic Fengyang Group. The Bagongshan Formation mainly consists of quartz sandstones. In places where the Caodian Formation is absent, the Bagongshan Formation sits directly on the Fengyang Group. The Liulaobei Formation mainly consists of shales and argillaceous limestones and preserves abundant organic-walled microfossils, including *Trachyhystrichosphaera*[14],

and macroscopic carbonaceous compression fossils, including *Chuaria*, *Tawuia*, *Sinosabellidites*, and *Anhuithrix*[15-17]. The Feishui Group represents another fining-upward cycle and is composed of the Shouxian, Jiuliqiao, and Sidingshan formations, in ascending order. The Shouxian Formation consists of sandstones with well-developed parallel and cross laminations. The Jiuliqiao Formation mainly consists of argillaceous limestones and stromatolitic limestones, bearing abundant carbonaceous compression macrofossils, i.e., *Chuaria*, *Tawuia*, *Sinosabellidites*, *Pararenicola*, and *Protoarenicola*[13,15,16]. The Jiuliqiao Formation was deposited in subtidal environment[13]. The Sidingshan Formation mainly consists of dolostones with chert nodules and columnar stromatolites, and is unconformably overlain by the Ediacaran Fengtai Formation or the Cambrian Houjiashan Formation.

The depositional age of the Huainan and Feishui groups is also constrained to be late Mesoproterozoic to early Neoproterozoic. Compiled youngest detrital zircon age populations[1] constrain the maximum depositional age of the upper Liulaobei Formation to be ~1000 Ma[18,19] and that of the Shouxian Formation to be ~950 Ma[18,20]. In combination with the organic-walled microfossil assemblage from the Liulaobei Formation (e.g., *Trachyhystrichosphaera*[14]) and the macroscopic carbonaceous compression fossils from the Jiuliqiao Formation e.g., *Chuaria*, *Tawuia*, *Sinosabellidites*, *Pararenicola*, and *Protoarenicola*[13]), the depositional age of the upper Liulaobei Formation can be constrained between ~1000 Ma and ~720 Ma, and that of the Shouxian and Jiuliqiao formations between ~950 Ma and ~720 Ma. Recently, Zhang et al.[21] reported an authigenic monazite SIMS Pb-Pb age of 1086 ± 120 Ma from the middle Liulaobei Formation which, given its large uncertainty, is consistent with a late Mesoproterozoic to early Tonian age of the lower to middle Liulaobei Formation.

Supplementary Figure

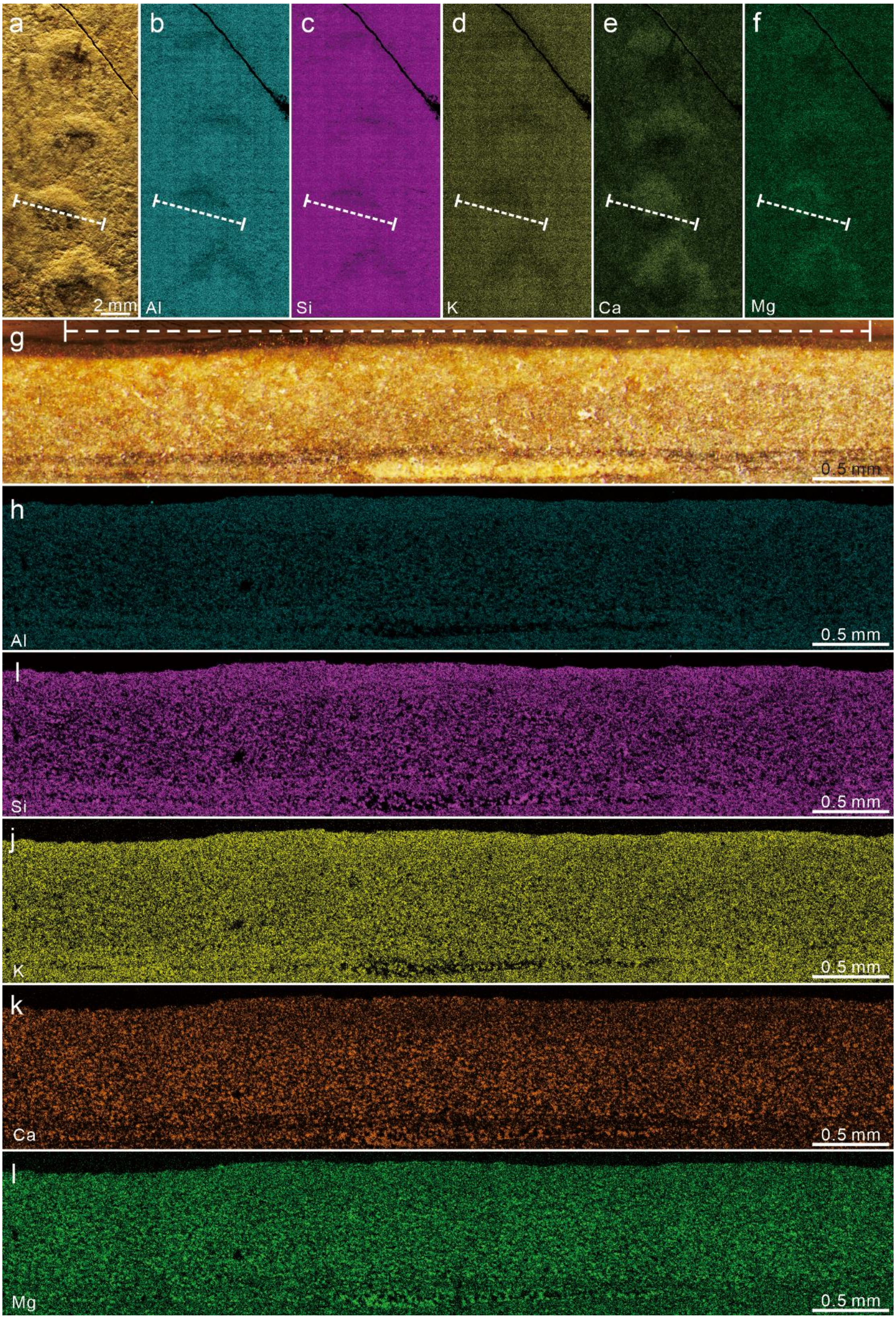

Supplementary Figure 1 | RLM images (a, g) and EDS elemental maps (b–f, h–l; element labeled in lower left) of a *Horodyskia moniliformis* specimen (SWZ-Z-53;

also illustrated in Figure 2f) from the Tonian Shiwangzhuang Formation. a–f Four beads with halos preserved; halos are enriched in calcium and magnesium, and slightly depleted in aluminum, silicon, and potassium, relative to the matrix and beads. **g–l** Side view of the bead in (**a**) cut perpendicular to bedding surface, with dashed line in (**g**) corresponding to dashed lines in (**a–f**); the uppermost fossil-bearing layer in (**g**) is darkened because of embedding resin (Technovit 7200 VLC); the halo shows no distinct difference from the underlying layer in aluminum, silicon, potassium, calcium, and magnesium enrichment.

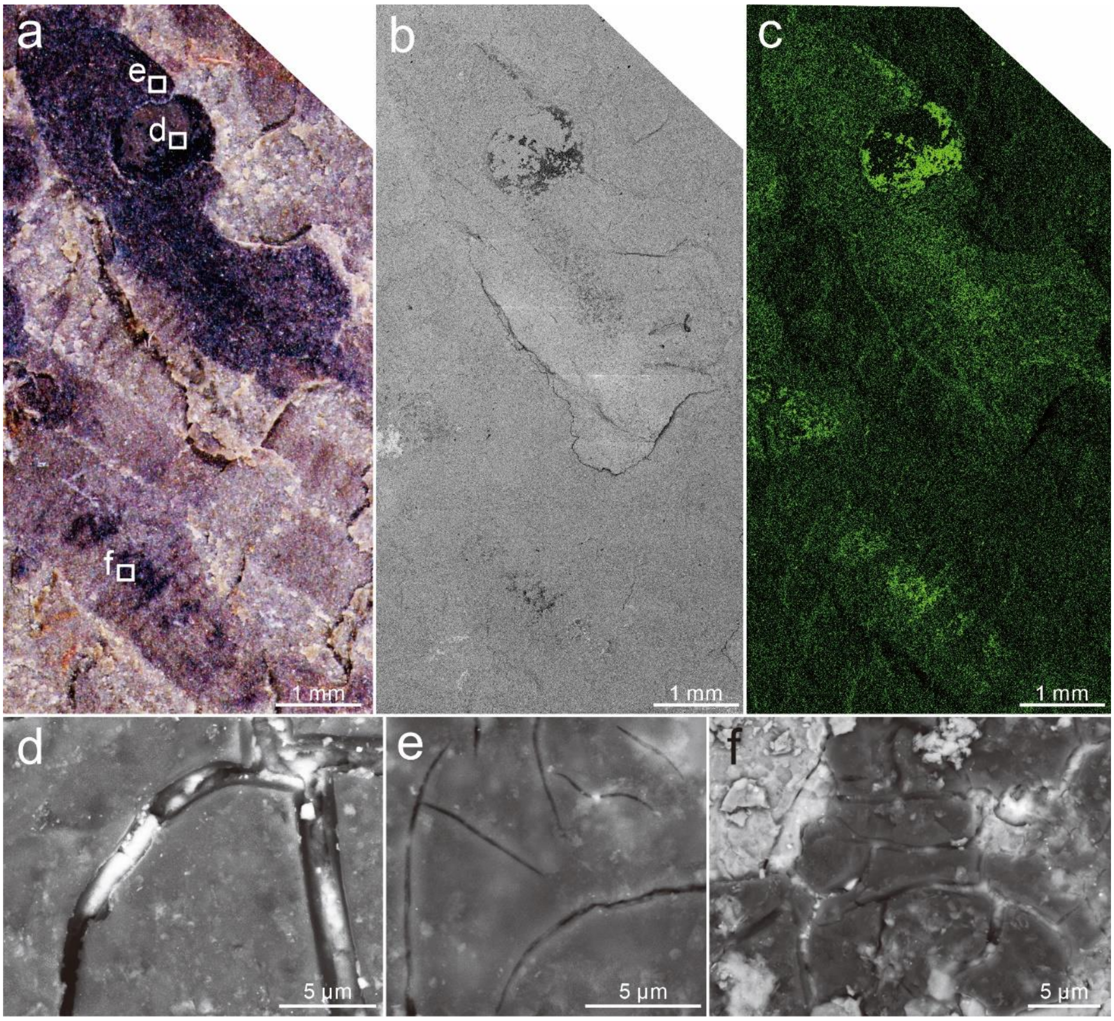

Supplementary Figure 2 | RLM image (a), BSE-SEM images (b, d–f), and EDS elemental map (c) of representative carbonaceous compression macrofossils from the Tonian Shiwangzhuang Formation. a–c RLM image (**a**), BSE-SEM image (**b**), and EDS elemental map (**c**) of three *Protoarenicola*/*Pararenicola* specimens with diagnostic transverse annulations (lower part) and a *Tawuia* specimen superimposed by a *Chuaria* specimen (upper part); the fossils are enriched in carbon (**c**); D29-5. **d–f** BSE images of box areas in (**a**), showing multiple cracks in carbonaceous films of the fossils.

Supplementary References

1 Pang, K. *et al.* Integrated Meso-Neoproterozoic stratigraphy in the Jiao-Liao-Xu-Huai area of North China Craton: a review. *J. Stratigr.* **45**, 467–492 (2021).

2 Peng, P. *et al.* Neoproterozoic (~ 900 Ma) Sariwon sills in North Korea: geochronology, geochemistry and implications for the evolution of the south-eastern margin of the North China Craton. *Gondwana Res.* **20**, 243–254 (2011).

3 Zhang, S., Zhao, Y., Ye, H. & Hu, G. Early Neoproterozoic emplacement of the diabase sill swarms in the Liaodong Peninsula and pre-magmatic uplift of the southeastern North China Craton. *Precambrian Res.* **272**, 203–225 (2016).

4 Wan, B. *et al.* Repositioning the Great Unconformity at the southeastern margin of the North China Craton. *Precambrian Res.* **324**, 1–17 (2019).

5 Li, G. *et al.* Organic-walled microfossils from the Tonian Tongjiazhuang Formation of the Tumen Group in western Shandong, North China Craton and their biostratigraphic significance. *Gondwana Res.* **76**, 260–289 (2019).

6 Li, G. *et al.* An assemblage of macroscopic and diversified carbonaceous compression fossils from the Tonian Shiwangzhuang Formation in western Shandong, North China. *Precambrian Res.* **346**, 105801 (2020).

7 Han, C. *et al.* First record of organic-walled microfossils from the Tonian Shiwangzhuang Formation of the Tumen Group in western Shandong, North China. *Palaeoworld* **30**, 208–219 (2021).

8 Bureau of Geology and Mineral Resources of Shandong Province. *Regional Geology of Shandong Province*. (Geological Publishing House, 1991).

9 Song, M. & Wang, P. *Regional Geology of Shandong Province*. (Map Publishing House of Shandong Province, 2003).

10 Hu, B. *et al.* Mesoproterozoic magmatic events in the eastern North China Craton and their tectonic implications: geochronological evidence from detrital zircons in the Shandong Peninsula and North Korea. *Gondwana Res.* **22**, 828–842 (2012).

11 Zhou, G. *et al.* Constraint of the depositional time of Tongjiazhuang Formation from LA-ICP-MS detrital zircon U-Pb age and microfossil assemblage. *J. Stratigr.* **43**,

229–242 (2019).
12 Lu, S., Xian, Z., Li, H., Wang, H. & Chu, H. Response of the North China Craton to Rodinia supercontinental events——GOSEN joining hypothesis. *Acta Geol. Sin.* **86**, 1396–1406 (2012).
13 Dong, L. *et al.* Restudy of the worm-like carbonaceous compression fossils *Protoarenicola*, *Pararenicola*, and *Sinosabellidites* from early Neoproterozoic successions in North China. *Palaeogeogr. Palaeoclimatol. Palaeoecol.* **258**, 138–161 (2008).
14 Tang, Q. *et al.* Organic-walled microfossils from the early Neoproterozoic Liulaobei Formation in the Huainan region of North China and their biostratigraphic significance. *Precambrian Res.* **236**, 157–181 (2013).
15 Sun, W., Wang, G. & Zhou, B. Macroscopic worm-like body fossils from the Upper Precambrian (900–700 Ma), Huainan district, Anhui, China and their stratigraphic and evolutionary significance. *Precambrian Res.* **31**, 377–403 (1986).
16 Wang, G., Zhou, B. & Xiao, L. Late Precambrian macrofossils from Huainan, Anhui and their significance. *J. Stratigr.* **8**, 271–278 (1984).
17 Pang, K. *et al.* Nitrogen-fixing heterocystous cyanobacteria in the Tonian Period. *Curr. Biol.* **28**, 616–622 (2018).
18 Li, G. *et al.* The characteristics of LA-ICP-MS detrital zircon U-Pb age from the Meso-Neoproterozoic strata in Huainan area and their geological significance. *J. Stratigr.* **45**, 115–141 (2021).
19 Li, J. *Analysis of sedimentary age, provenance and tectonic background of Fengyang group and Bagongshan group of Precambrian in Fengyang area* master thesis, Hefei University of Technology (2019).
20 Zhao, H. *et al.* New geochronologic and paleomagnetic results from early Neoproterozoic mafic sills and late Mesoproterozoic to early Neoproterozoic successions in the eastern North China Craton, and implications for the reconstruction of Rodinia. *Geol. Soc. Am. Bull.* **132**, 739–766 (2020).
21 Zhang, S. *et al.* SIMS Pb-Pb dating of phosphates in the Proterozoic strata of SE North China Craton: constraints on eukaryote evolution. *Precambrian Res.* **371**, 106562

(2022).
