# Supplementary material for: Tonian carbonaceous compressions indicate that Horodyskia is one of the oldest multicellular and coenocytic macro-organisms
Source: Commun Biol. 2023 Apr 12;6:399. doi: 10.1038/s42003-023-04740-2 (PMC10097871; doi:10.1038/s42003-023-04740-2)
Supplement: Supplementary file 3 — Description of Additional Supplementary Files [file 42003_2023_4740_MOESM3_ESM.pdf]

Description of Additional Supplementary Files

File name: Supplementary Data 1

Description: The source data behind Figure 6 in the paper.

File name: Supplementary Data 2

Description: The source data behind Figure 7 in the paper.

File name: Supplementary Data 3

Description: The catalog numbers for the specimens in the paper.
