# Supplementary material for: Tonian carbonaceous compressions indicate that Horodyskia is one of the oldest multicellular and coenocytic macro-organisms
Source: Commun Biol. 2023 Apr 12;6:399. doi: 10.1038/s42003-023-04740-2 (PMC10097871; doi:10.1038/s42003-023-04740-2)
Supplement: Supplementary file 7 — Reporting Summary [file 42003_2023_4740_MOESM7_ESM.pdf]

nature portfolio

Corresponding author(s): Ke Pang

Last updated by author(s): March 13th, 2023

Reporting Summary

Nature Portfolio wishes to improve the reproducibility of the work that we publish. This form provides structure for consistency and transparency in reporting. For further information on Nature Portfolio policies, see our Editorial Policies and the Editorial Policy Checklist.

Statistics

For all statistical analyses, confirm that the following items are present in the figure legend, table legend, main text, or Methods section.

n/a	Confirmed	
☐	☒	The exact sample size (*n*) for each experimental group/condition, given as a discrete number and unit of measurement
☐	☒	A statement on whether measurements were taken from distinct samples or whether the same sample was measured repeatedly
☒	☐	The statistical test(s) used AND whether they are one- or two-sided<br>*Only common tests should be described solely by name; describe more complex techniques in the Methods section.*
☒	☐	A description of all covariates tested
☒	☐	A description of any assumptions or corrections, such as tests of normality and adjustment for multiple comparisons
☐	☒	A full description of the statistical parameters including central tendency (e.g. means) or other basic estimates (e.g. regression coefficient) AND variation (e.g. standard deviation) or associated estimates of uncertainty (e.g. confidence intervals)
☒	☐	For null hypothesis testing, the test statistic (e.g. *F*, *t*, *r*) with confidence intervals, effect sizes, degrees of freedom and *P* value noted<br>*Give P values as exact values whenever suitable.*
☒	☐	For Bayesian analysis, information on the choice of priors and Markov chain Monte Carlo settings
☒	☐	For hierarchical and complex designs, identification of the appropriate level for tests and full reporting of outcomes
☒	☐	Estimates of effect sizes (e.g. Cohen's *d*, Pearson's *r*), indicating how they were calculated

Our web collection on statistics for biologists contains articles on many of the points above.

Software and code

Policy information about availability of computer code

Data collection	LabSpec 6.0; Image J
Data analysis	PeakFit 4.2; JMP13; Microsoft Excel 2010

For manuscripts utilizing custom algorithms or software that are central to the research but not yet described in published literature, software must be made available to editors and reviewers. We strongly encourage code deposition in a community repository (e.g. GitHub). See the Nature Portfolio guidelines for submitting code & software for further information.

Data

Policy information about availability of data

All manuscripts must include a data availability statement. This statement should provide the following information, where applicable:

- Accession codes, unique identifiers, or web links for publicly available datasets
- A description of any restrictions on data availability
- For clinical datasets or third party data, please ensure that the statement adheres to our policy

All data needed to evaluate the conclusions in the paper are present in the paper and its supplementary information files, or from the corresponding authors upon reasonable request. Specimens from the Shiwangzhuang Formation illustrated in this paper are reposited and available at College of Earth Science and Engineering, Shandong University of Science and Technology and specimens from the Jiuliqiao Formation illustrated in this paper are reposited and available at Nanjing Institute

of Geology and Palaeontology, Chinese Academy of Sciences (NIGPAS). Source data for Figures 6 and 7are provided with this paper as Supplementary Data 1 and 2. The catalog numbers for the specimens are provided in Supplementary Data 3.

Human research participants

Policy information about studies involving human research participants and Sex and Gender in Research.

Reporting on sex and gender	*Use the terms sex (biological attribute) and gender (shaped by social and cultural circumstances) carefully in order to avoid confusing both terms. Indicate if findings apply to only one sex or gender; describe whether sex and gender were considered in study design whether sex and/or gender was determined based on self-reporting or assigned and methods used. Provide in the source data disaggregated sex and gender data where this information has been collected, and consent has been obtained for sharing of individual-level data; provide overall numbers in this Reporting Summary. Please state if this information has not been collected. Report sex- and gender-based analyses where performed, justify reasons for lack of sex- and gender-based analysis.*
Population characteristics	*Describe the covariate-relevant population characteristics of the human research participants (e.g. age, genotypic information, past and current diagnosis and treatment categories). If you filled out the behavioural & social sciences study design questions and have nothing to add here, write "See above."*
Recruitment	*Describe how participants were recruited. Outline any potential self-selection bias or other biases that may be present and how these are likely to impact results.*
Ethics oversight	*Identify the organization(s) that approved the study protocol.*

Note that full information on the approval of the study protocol must also be provided in the manuscript.

Field-specific reporting

Please select the one below that is the best fit for your research. If you are not sure, read the appropriate sections before making your selection.

☐ Life sciences ☐ Behavioural & social sciences ☒ Ecological, evolutionary & environmental sciences

For a reference copy of the document with all sections, see nature.com/documents/nr-reporting-summary-flat.pdf

Ecological, evolutionary & environmental sciences study design

All studies must disclose on these points even when the disclosure is negative.

Study description	Palaeontological study of Horodyskia specimens mainly preserved as carbonaceous compressions or organic-walled macrofossils from the Tonian Shiwangzhuang Formation (~850–720 Ma) in western Shandong and Jiuliqiao Formation (~950–720 Ma) in Huainan region, North China. Total number of measured specimens/strings was 87. Measurement were repeated 3 times.
Research sample	Specimens were collected from an argillaceous limestone horizon ca. 74 m below the top of the Shiwangzhuang Formation (~850–720 Ma) at the Baishicun section (36°30′39″N, 119°07′39″E), Anqiu region, western Shandong Province, North China (Fig. 1b) and an argillaceous limestone horizon at ca. 14 m below the top of Jiuliqiao Formation at the Baiguashan section (32°44′24″N, 117°11′24″E), Huainan region, northern Anhui Province, North China (Fig. 1c). All illustrated specimens from the Shiwangzhuang Formation are deposited in College of Earth Science and Engineering, Shandong University of Science and Technology and illustrated specimens from the Jiuliqiao Formation are deposited at Nanjing Institute of Geology and Palaeontology, Chinese Academy of Sciences.
Sampling strategy	Fossils were collected from outcrops with stratigraphic horizon noted. A total of 46 Horodyskia moniliforms specimens and 41 Horodyskia minor specimens were measured. This sample size is deemed sufficient in paleontological investigations. No sample size calculation was performed.
Data collection	Measurements were made on photographs using Image J.
Timing and spatial scale	Specimens were collected in 2020-2022 from an argillaceous limestone horizon ca. 74 m below the top of the Shiwangzhuang Formation (~850–720 Ma) at the Baishicun section (36°30′39″N, 119°07′39″E), Anqiu region, western Shandong Province, North China and an argillaceous limestone horizon at ca. 14 m below the top of Jiuliqiao Formation at the Baiguashan section (32°44′24″N, 117°11′24″E), Huainan region, northern Anhui Province, North China.
Data exclusions	No data were excluded from analysis.
Reproducibility	To ensure reproducibility, details about fossil locality and stratigraphic horizon have been noted.
Randomization	None.
Blinding	None.

Did the study involve field work? ☒ Yes ☐ No

Field work, collection and transport

Field conditions	The field site is located in the temperate zone. Climate is cool in the field season (autumn time). Outcrops are well exposed. Excavation was required to remove slab of fossil specimens.
Location	Located at the Baishicun section (36°30′39″N, 119°07′39″E), Anqiu region, western Shandong Province, North China and at the Baiguashan section (32°44′24″N, 117°11′24″E) , Huainan region, northern Anhui Province, North China
Access & import/export	Collection of fossil specimens was carried out in a responsible manner and in compliance with the local, national and international laws. All illustrated specimens from the Shiwangzhuang Formation are deposited in College of Earth Science and Engineering, Shandong University of Science and Technology and illustrated specimens from the Jiuliqiao Formation are deposited at Nanjing Institute of Geology and Palaeontology, Chinese Academy of Sciences.
Disturbance	None.

Reporting for specific materials, systems and methods

We require information from authors about some types of materials, experimental systems and methods used in many studies. Here, indicate whether each material, system or method listed is relevant to your study. If you are not sure if a list item applies to your research, read the appropriate section before selecting a response.

Materials & experimental systems

n/a	Involved in the study	
☒	☐	Antibodies
☒	☐	Eukaryotic cell lines
☐	☒	Palaeontology and archaeology
☒	☐	Animals and other organisms
☒	☐	Clinical data
☒	☐	Dual use research of concern

Methods

n/a	Involved in the study	
☒	☐	ChIP-seq
☒	☐	Flow cytometry
☒	☐	MRI-based neuroimaging

Palaeontology and Archaeology

Specimen provenance	Specimens were collected from an argillaceous limestone horizon ca. 74 m below the top of the Shiwangzhuang Formation (~850–720 Ma) at the Baishicun section (36°30′39″N, 119°07′39″E), Anqiu region, western Shandong Province, North China and an argillaceous limestone horizon at ca. 14 m below the top of Jiuliqiao Formation at the Baiguashan section (32°44′24″N, 117°11′24″E), Huainan region, northern Anhui Province, North China.
Specimen deposition	All illustrated specimens from the Shiwangzhuang Formation are deposited in College of Earth Science and Engineering, Shandong University of Science and Technology and illustrated specimens from the Jiuliqiao Formation are deposited at Nanjing Institute of Geology and Palaeontology, Chinese Academy of Sciences.
Dating methods	The youngest detrital zircon age from the Fulaishan Formation and macroscopic carbonaceous compression fossils from the Shiwangzhuang Formation, including Chuaria, Tawuia, Sinosabellidites, Protoarenicola, and Pararenicola, constrain the depositional age of the Fulaishan and Shiwangzhuang formations to be ~850–720 Ma.<br>Compiled youngest detrital zircon age populations constrain the maximum depositional age of the Shouxian and Jiuliqiao formations to be ~950 Ma; In combination with the macroscopic carbonaceous compression fossils from the Jiuliqiao Formation [e.g., Chuaria, Tawuia, Sinosabellidites, Pararenicola, and Protoarenicola], the depositional age of the Shouxian and Jiuliqiao formations between ~950 Ma and ~720 Ma.

☒ Tick this box to confirm that the raw and calibrated dates are available in the paper or in Supplementary Information.

Ethics oversight	No ethical approval or guidance was required for this palaeontological study.

Note that full information on the approval of the study protocol must also be provided in the manuscript.
